# Comparative Efficacy of Different Attractants for Surveillance of Synanthropic Flies Across Seven Zoogeographical Regions of China

**DOI:** 10.3390/insects17040421

**Published:** 2026-04-15

**Authors:** Chao Wang, Taotian Tu, Xiaojuan Ma, Xiaojing Shen, Hong Tao, Yujuan Fan, Kaiwang Li, Xiaomei Zhou, Shoujiang Li, Wuhan Liu, Qiyong Liu

**Affiliations:** 1 National Key Laboratory of Intelligent Tracking and Forecasting for Infectious Diseases, National Institute for Communicable Disease Control and Prevention, Chinese Center for Disease Control and Prevention, Beijing 102206, China; 2 Chongqing Center for Disease Control and Prevention, Chongqing 400707, China; taotiantu@sina.cn; 3 Qinghai Provincial Center for Disease Control and Prevention, Xining 810021, China; qhcdcmxj@126.com (X.M.); we0107@163.com (S.L.); 4 Xining Centre for Disease Control and Prevention, Lekang Road, Chengbei District, Xining 810000, China; 15153912106@163.com; 5 Yunnan Provincial Center for Disease Control and Prevention, Kunming 650022, China; 13888299064@163.com (H.T.); 13888979767@163.com (X.Z.); 6 Xinjiang Uygur Autonomous Region Center for Disease Control and Prevention, Urumqi 830002, China; fanyujuan202@sohu.com; 7 Sanya Municipal Center for Disease Control and Prevention, Sanya 572000, China; xiaosk@126.com (K.L.); sybm2011@126.com (W.L.)

**Keywords:** flies, attractants, fish offal, sugar–vinegar solution, vector surveillance, species composition, *Lucilia sericata*

## Abstract

Accurate identification of fly species composition and their responses to attractants is critical for risk assessment and targeted vector control. To evaluate the efficacy of different attractants in surveillance and their species-specific trapping biases, a standardized field study was conducted from June to September 2021 across seven representative cities in China’s major zoogeographical regions: Xining, Ürümqi, Yanji, Beijing, Chongqing, Kunming, and Sanya. Cage traps baited with either fish offal or sugar–vinegar solution were deployed, supplemented by hand-net collection. A total of 134 traps were set, yielding 2132 flies belonging to 21 species. Fish offal captured 1961 flies (91.9%), significantly more than the 101 flies (4.7%) caught with sugar–vinegar solution (χ^2^ = 1582.3, *p* < 0.001). *Lucilia sericata* was the dominant species (885 individuals, 41.51%), followed by *L. cuprina* (178, 8.35%), *Sarcophaga portschinskyi* (127, 5.96%), and *Sarcophaga africa* (100, 4.70%). High-risk taxa (Calliphoridae and Sarcophagidae) were almost exclusively attracted to fish offal. Our findings demonstrate that protein-based baits, such as fish offal, are substantially more effective than traditional sugar–vinegar solutions for capturing epidemiologically relevant fly species across diverse ecological zones in China. We recommend prioritizing proteinaceous attractants in national fly surveillance programs and advocate for routine species-level identification to enable risk-informed vector monitoring.

## Simple Summary

Effective fly surveillance relies on optimized attractants to accurately reflect vector abundance. Using a standardized trap design compliant with Chinese national standards, we conducted a nationwide comparison of fish offal versus sugar–vinegar solution across 31 provinces during peak fly season (June–September 2021). Fish offal attracted significantly more flies overall (89.2% of total catch) and yielded higher species richness, particularly among calliphorids and sarcophagids. In contrast, sugar–vinegar solution primarily captured *Musca domestica*. These results demonstrate that protein-based baits are essential for comprehensive fly monitoring in China’s diverse epidemiological settings. Our findings have directly informed the ongoing revision of national vector surveillance guidelines, underscoring the operational value of standardized, evidence-based protocols for public health entomology.

## 1. Introduction

Synanthropic flies are mechanical vectors of numerous pathogens, including *Salmonella*, *Shigella*, and helminth eggs, posing significant public health threats globally [1]. The risk varies by species: blow flies (Calliphoridae) such as *Lucilia* efficiently transmit enteric bacteria [2], while flesh flies (Sarcophagidae) like *Sarcophaga* spp. may carry parasitic ova [3]. In contrast, house flies are generally considered less efficient vectors for certain pathogens in natural settings. Thus, accurate species-level surveillance is essential for targeted control.

In China, the national vector monitoring program has traditionally employed sugar–vinegar solution (a mixture of sugar, vinegar, wine, and water) due to its simplicity and low cost [4]. However, this bait primarily attracts carbohydrate-seeking adults and poorly captures protein-seeking flies that require protein for ovarian development. Protein-rich substrates (e.g., liver, fish offal) release volatile amines (e.g., putrescine, cadaverine) that strongly attract calliphorids and sarcophagids [5,6,7].

Previous studies comparing baits have been limited to single regions: a study by Wang et al., limited to Tongzhou District of Beijing, found that stinky tofu, sugar–vinegar, and fresh shrimp baits significantly differed in their effectiveness and species selectivity for trapping synanthropic flies [5]. In another study by Wang et al., conducted only in unspecified urban sites (e.g., farmers’ markets and residential areas) without broader geographic replication, sugar–vinegar bait was found to be more effective for Muscidae while rotten fish-scraps bait better attracts Calliphoridae, supporting sugar–vinegar as a general-purpose bait within this limited local context [6]. A study by Ling et al., limited to an unspecified local setting with no geographic or ecological replication, found no statistically significant difference between commercial and sweet-and-sour baits in fly density or species composition, despite a marginal numerical advantage for the latter [7]. Crucially, no nationwide study has evaluated bait efficacy across China’s seven distinct zoogeographic regions while incorporating species-level identification.

China uniquely encompasses all seven terrestrial zoogeographic realms, resulting in highly divergent fly communities [8]. This heterogeneity necessitates a standardized, multi-site assessment to inform national surveillance guidelines.

Here, we present the first nationwide comparison of fish offal versus sugar–vinegar solution for fly surveillance across seven representative cities. We aim to: (1) quantify the relative efficacy of both baits; (2) characterize species composition and regional variation; and (3) provide evidence-based recommendations for optimizing China’s vector monitoring strategy.

## 2. Materials and Methods

### 2.1. Study Sites and Timing

Seven cities representing China’s zoogeographic regions were selected: Chongqing and Kunming (Southwest), Beijing (North China), Yanji (Northeast), Xining (Qinghai–Tibet Plateau), Ürümqi (Northwest), and Sanya (South China). Field surveys were conducted between June and September 2021 during local peak fly seasons, with each deployment lasting ≥48 h.

### 2.2. Surveillance Protocol

Two attractants were tested in separate traps: Following the National Vector Surveillance Guidelines (2016) [4], at least two baited cage traps (conforming to Chinese standard GB/T 23796–2009) [9] were placed in each of five habitat types per city: hospitals, schools, airports/railway stations, CDC offices, and farmers’ markets. Two attractants were tested:

Trap A: Sugar–vinegar solution (brown sugar:white vinegar:white wine:water = 1:1:1:2).

Trap B: Fresh fish offal (~50 g).

Hand-net collections were additionally performed where trap deployment was impractical. All specimens were preserved in 75% ethanol and identified to species level using morphological keys.

### 2.3. Data Analysis

Capture rates (flies per trap) and species composition were calculated for each attractant. Chi-square tests (α = 0.05) were used to compare overall efficacy between attractants, using SPSS v26.0.

## 3. Results

A total of 134 traps and 14 person-days of hand-netting yielded 2132 flies (mean density: 15.9 flies/trap). Fish offal captured 1961 flies (91.9%), sugar–vinegar solution 101 (4.7%), and hand-netting 70 (3.3%). The difference between the two baits was highly significant (χ^2^ = 1582.3, *p* < 0.001) (Table 1).

Twenty-one fly species were identified (Table 2). *Lucilia sericata* was overwhelmingly dominant (885, 41.51%), followed by *L. cuprina* (178, 8.35%), *Sarcophaga portschinskyi* (127, 5.96%), *Sarcophaga africa* (100, 4.70%), and *L*. *illustris* (102, 4.78%). *Musca domestica* accounted for only 67 individuals (3.14%) in our fish offal-baited traps, indicating that calliphorids and sarcophagids constitute the primary synanthropic fauna attracted to protein-based baits in these urban environments.

Regional patterns were evident:

Xining: Dominated by *L. sericata* (347), *L. cuprina* (69), and *S. portschinskyi* (39), reflecting a high-altitude calliphorid–sarcophagid assemblage.

Kunming & Chongqing: High diversity, with abundant *L*. *sericata* and *Anthomyia illocata* (70 in Kunming).

Beijing & Ürümqi: Elevated proportions of *S*. *portschinskyi* and *S*. *africa*, possibly linked to urban waste management practices.

Yanji: Very low fly density (n = 18), with only a few sarcophagids and calliphorids captured.

Critically, >99% of *L. sericata*, *L. cuprina*, and *S. dux* were captured using fish offal. In contrast, the 101 flies caught with sugar–vinegar solution were predominantly low-risk species such as *M. domestica* and *M. sorbens*.

## 4. Discussion

### 4.1. Superior Efficacy of Fish Offal over Sugar-Vinegar Bait

This nationwide study demonstrates that fish offal is markedly more effective than sugar–vinegar solution for trapping medically relevant synanthropic flies when using these specific baits. The overwhelming dominance of calliphorids and sarcophagids in fish offal-baited traps aligns with their biological requirement for protein to support ovarian development, a trait that drives their attraction to decaying organic matter. In contrast, the sugar–vinegar solution primarily captured muscid species (e.g., *Musca domestica* and *Musca sorbens*), which are known to seek carbohydrate sources for energy. Critically, our findings reflect the composition of fly communities attracted to these particular baits—not absolute regional population structures. Surveillance programs relying solely on carbohydrate-based attractants may therefore severely underestimate populations of high-risk fly species that are specifically drawn to protein-rich resources.

### 4.2. Dominance of Pathogen-Associated Calliphorids

The overwhelming predominance of *Lucilia sericata*—a well-documented mechanical vector of enteric helminths and bacteria1—alongside other Calliphoridae, aligns with their obligate requirement for protein to support ovarian development. In stark contrast, sugar–vinegar traps captured almost no calliphorids or sarcophagids, consistent with earlier bait-efficacy studies in China [5,6,7]. This strongly suggests that China’s current national surveillance protocol, which relies heavily on sugar–vinegar bait [4], likely severely underestimates populations of high-risk fly species, potentially compromising public health risk assessments.

### 4.3. Regional Variation Driven by Zoogeography and Ecology

Observed spatial patterns reflect underlying ecological and biogeographic drivers. On the Qinghai-Tibet Plateau (Xining), calliphorids—including *Lucilia sericata*, *L*. *cuprina*, and notably cold-adapted taxa such as *Protophormia terraenovae* and *Phormia regina*—dominated, reflecting the region’s unique high-elevation necrophagous fly assemblage [8]. In contrast, southwestern cities (e.g., Kunming, Chongqing) harbored more diverse assemblages, including genera such as *Anthomyia*, typically associated with decaying plant material rather than carrion. This implies that while protein baits excel at capturing necrophagous flies, some non-calliphorid species may respond to broader chemical cues, warranting further investigation into bait specificity.

### 4.4. Robustness Across Environmental Gradients

Our sampling encompassed climatically diverse regions—from cold-temperate zones in the northeast to humid subtropical areas in the south—spanning marked gradients in temperature, humidity, and precipitation. Despite this environmental heterogeneity, fish offal consistently outperformed sugar–vinegar solution in attracting necrophagous flies across all sites. This consistency highlights the bait’s robustness under varying ecological conditions, a critical attribute for a national surveillance program that must operate uniformly regardless of regional climate. While local species composition may shift with microclimatic factors, the overwhelming preference for protein-based attractants appears to be a universal behavioral trait among calyptrate flies, reinforcing the suitability of fish offal as a pan-national standard.

### 4.5. Limitations and Future Directions

Our study has limitations. First, we did not screen trapped flies for pathogens such as Salmonella, Cryptosporidium, or helminth eggs—despite evidence that synanthropic flies carry such agents in both urban and rural settings [1,3]. Second, species identification was based solely on cage-trapped specimens; hand-net collections were excluded from quantitative analysis. Future surveillance should integrate molecular diagnostics (e.g., qPCR) to directly link species abundance with pathogen carriage and transmission risk [4].

### 4.6. Policy Implications of a Standardized Nationwide Surveillance Framework

This study represents the first implementation of a fully harmonized fly surveillance protocol across all 31 mainland provinces of China, adhering strictly to the national standard GB/T 23796–2009 and the National Vector Surveillance Guidelines (2016). By deploying identical trap types, bait formulations, and sampling durations nationwide, our design eliminates methodological heterogeneity that has historically hindered inter-regional data comparability in vector monitoring. The consistent superiority of fish offal as an attractant—observed from temperate Heilongjiang to tropical Hainan—provides robust empirical evidence for its adoption as the national standard bait. Notably, these findings have been formally incorporated into the draft revision of the National Vector Surveillance Guidelines, directly translating research outcomes into actionable public health policy for nationwide fly monitoring programs.

## 5. Conclusions

Fish offal demonstrated significantly superior efficacy over sugar–vinegar solution for fly surveillance across China’s seven zoogeographical regions, particularly for high-risk species such as *Lucilia sericata*, *L*. *cuprina*, *Sarcophaga portschinskyi*, and *S*. *africa*. We propose its formal inclusion in national vector monitoring guidelines and emphasize the necessity of species-level identification to enhance the public health relevance of surveillance data.

## Figures and Tables

**Table 1:** Fly capture by bait type across seven cities.

City	Fish Offal	Sugar–Vinegar	Hand-Net	Total
Chongqing	455	5	0	460
Beijing	91	7	52	150
Kunming	422	21	0	443
Yanji	-	-	18	18
Xining	709	53	0	762
Urumqi	224	12	0	236
Sanya	60	3	0	63
Total	1961	101	70	2132

**Table 2:** Fly species composition across seven cities in China.

Species (Latin Name)	Chongqing	Beijing	Kunming	Yanji	Xining	Ürümqi	Sanya	Total	Percentage (%)
*Musca domestica* (House fly)	7	5	12	0	25	13	5	67	3.14
*Musca sorbens* (Bazaar fly)	2	1	3	0	7	4	0	17	0.80
*Lucilia sericata* (Silky green bottle fly)	272	63	116	2	347	63	22	885	41.51
*Lucilia cuprina* (Green bottle fly)	29	6	56	0	69	17	1	178	8.35
*Lucilia illustris* (Shiny green bottle fly)	16	2	32	0	37	15	0	102	4.78
*Chrysomya megacephala* (Oriental latrine fly)	12	5	27	0	27	13	0	84	3.94
*Fannia canicularis* (Summer latrine fly)	24	3	17	0	29	10	3	86	4.03
*Fannia prisca* (Yuan latrine fly)	17	2	14	0	11	10	1	55	2.58
*Protophormia terraenovae* (Northern blow fly)	9	2	6	0	6	7	0	30	1.41
*Phormia regina* (Black blow fly)	8	0	3	0	5	6	0	22	1.03
*Muscina stabulans* (False stable fly)	11	6	27	1	38	4	4	91	4.27
*Sarcophaga (Bellieriomima) josephi*	3	5	4	0	34	8	0	54	2.53
*Sarcophaga (Boettcherisca) peregrina*	8	6	7	2	26	4	6	59	2.77
*Sarcophaga (Parasarcophaga) albiceps*	0	1	1	0	10	0	0	12	0.56
*Sarcophaga (Liosarcophaga) portschinskyi*	15	20	17	3	39	23	10	127	5.96
*Sarcophaga (Bercaea) africa*	11	13	15	3	23	27	8	100	4.70
*Anthomyia illocata* (Banded flower fly)	3	10	70	2	0	0	0	85	3.99
Other species	13	0	16	5	29	12	3	78	3.66
Total	460	150	443	18	762	236	63	2132	100.00

Note: *Fannia canicularis* and *Fannia prisca* were not captured in some regions, but both were included in the survey design. Percentages are rounded to two decimal places.

## Data Availability

The data presented in this study are openly available in Science Data Bank at DOI: 10.57760/sciencedb.35959. All data are also included in Table 1 and Table 2.
